# Novel Histone Deacetylase Class IIa Selective Substrate Radiotracers for PET Imaging of Epigenetic Regulation in the Brain

**DOI:** 10.1371/journal.pone.0133512

**Published:** 2015-08-05

**Authors:** Robin Bonomi, Uday Mukhopadhyay, Aleksandr Shavrin, Hsien-Hsien Yeh, Anjoy Majhi, Sajeewa W. Dewage, Amer Najjar, Xin Lu, G. Andrés Cisneros, William P. Tong, Mian M. Alauddin, Ren-Shuan Liu, Thomas J. Mangner, Nashaat Turkman, Juri G. Gelovani

**Affiliations:** 1 Department of Biomedical Engineering, Wayne State University, Detroit, MI 48202, United States of America; 2 Positron Emission Tomography Center, Wayne State University, Detroit, MI 48202, United States of America; 3 Department of Chemistry, Wayne State University, Detroit, MI 48202, United States of America; 4 Department of Cancer Systems Imaging, University of Texas MD Anderson Cancer Center, Houston, TX 77030, United States of America; 5 Center for Advanced Biomedical Imaging, University of Texas MD Anderson Cancer Center, Houston, TX 77030, United States of America; 6 National Cyclotron and Radiochemistry Center, National Yang Ming University, Taipei, Taiwan; 7 Department of Biomedical Imaging and Radiological Sciences, National Yang Ming University, Taipei, Taiwan; Stanford University School of Medicine, UNITED STATES

## Abstract

Histone deacetylases (HDAC’s) became increasingly important targets for therapy of various diseases, resulting in a pressing need to develop HDAC class- and isoform-selective inhibitors. Class IIa deacetylases possess only minimal deacetylase activity against acetylated histones, but have several other client proteins as substrates through which they participate in epigenetic regulation. Herein, we report the radiosyntheses of the second generation of HDAC class IIa–specific radiotracers: 6-(di-fluoroacetamido)-1-hexanoicanilide (DFAHA) and 6-(tri-fluoroacetamido)-1-hexanoicanilide ([^18^F]-TFAHA). The selectivity of these radiotracer substrates to HDAC class IIa enzymes was assessed *in vitro*, in a panel of recombinant HDACs, and *in vivo* using PET/CT imaging in rats. [^18^F]TFAHA showed significantly higher selectivity for HDAC class IIa enzymes, as compared to [^18^F]DFAHA and previously reported [^18^F]FAHA. PET imaging with [^18^F]TFAHA can be used to visualize and quantify spatial distribution and magnitude of HDAC class IIa expression-activity in different organs and tissues *in vivo*. Furthermore, PET imaging with [^18^F]TFAHA may advance the understanding of HDACs class IIa mediated epigenetic regulation of normal and pathophysiological processes, and facilitate the development of novel HDAC class IIa-specific inhibitors for therapy of different diseases.

## Introduction

There is an increasing demand for understanding the molecular pathophysiology of epigenetic regulation by histone deacetylases (HDACs). To date, 18 HDAC enzymes have been identified in humans and are typically divided into four major classes [1]. Class I HDACs includes: HDAC1, HDAC2, HDAC3, and HDAC8, which have significant domain and sequence similarity. HDAC’s in this class have typically a nuclear localization [2] and are involved in the regulation of cellular proliferative activity [3,4]. Class II HDACs, including HDAC4, HDAC5, HDAC6, HDAC7, HDAC9, and HDAC10, shuttle between the cytoplasm and nucleus. Class II is divided further into Class IIa (HDACs 4, 5, 7, 9) and Class IIb (HDACs 6, 10), with the primary difference in the central domain of class IIb, containing a zinc finger motif, while class IIa contains a structural regulatory zinc-binding domain [5,6]. Class IIa is able to shuttle more readily between the nucleus and the cytoplasm, whereas class IIb is primarily localized in the cytoplasm. In studying brain activity and pathophysiology, class IIa HDACs are more relevant over class IIb due to their increased expression in certain structures of the brain. HDAC enzymes in classes I, II, and IV are dependent on Zn^2+^ for enzymatic activity. Class III is comprised of HDAC enzymes termed “silent information regulators” or “sirtuins” (SIRTs), which represent a family of closely related deacetylases that are Zn^2+^-independent but are dependent on NAD^+^. Seven sub-types of SIRTs (SIRT 1–7) have been identified in humans [1]. SIRT enzymes are becoming increasingly scrutinized for their involvement in cancer, mental illness, and lifespan regulation. Class IV HDACs is comprised of a single HDAC protein, HDAC11, which is considered to be separate from classes I and II due to its biological actions [7,8]. The mechanisms of catalytic activity amongst different HDAC classes and unique structures of individual isoforms correspond to peptide sequence-specificity and types of post-translational modifications of their client proteins (i.e., acetylation, succinylation, myristoylation, etc.) [9]. This allows for the development of class and isoform-specific inhibitors for treatment of various pathological conditions and diseases [10].

Longitudinal studies in experimental animal models and in humans, aimed to investigate different HDACs-mediated epigenetic mechanisms, could be greatly facilitated by the availability of agents for non-invasive, repetitive, and quantitative imaging of enzyme expression-activity *in vivo*. Previously, we demonstrated that Boc-Lys-trifluoroacetate (BLT) could be used for ^19^F-NMR spectroscopic monitoring of HDAC activity *in vivo* [11,12]. Also, our group developed the first radiotracer for PET imaging of HDAC expression and activity, the 6-([^18^F]fluoroacetamido)-1-hexanoicanilide, termed [^18^F]FAHA [13]. We demonstrated that after intravenous injection, [^18^F]FAHA rapidly accumulates in the brain in rats and in rhesus macaques, and that the rate of [^18^F]FAHA accumulation in the brain is inhibited in a dose-dependent manner by HDAC inhibitor SAHA (vorinostat) [14–16]. Other investigators confirmed the results of our initial studies using PET imaging with [^18^F]FAHA in mice [17] and baboons [18,19]. These studies reproducibly demonstrated accumulation of [^18^F]FAHA-derived radioactivity in the *n*. *accumbens*, *amygdala*, *hippocampus*, *periaqueductal grey matter* and in the *cerebellum*. These structures of the brain have selective upregulation of HDACs class IIa expression, as demonstrated by immunohistochemical staining methods in rhesus macaque [14–16] and human brain [20]. Furthermore, the importance of HDAC IIa enzymes in *n*. *accumbens*, such as HDAC4 and HDAC5, has been demonstrated in addiction, specifically in the development of drug-seeking behavior [21]. Evidence from different laboratories using cell culture and in vivo model systems indicates that HDAC4 plays an essential role in the development of central nervous system [22] and in maintenance of neuronal survival [23,24]. The HDAC4 null knockout mice, which die within 2 weeks of birth, display cerebellar degeneration [25]. Also, selective deletion of *Hdac4* in the brain resulted in loss of learning and memory function [26], and haploinsufficiency of *HDAC4* in humans is associated with brachydactyly mental retardation syndrome [27].

Considering the importance of HDAC class IIa in epigenetic regulatory mechanisms involved in brain development and function, we developed the second generation of HDAC class IIa–specific radiotracers: 6-(di-fluoroacetamido)-1-hexanoicanilide (DFAHA) and 6-(tri-fluoroacetamido)-1-hexanoicanilide ([^18^F]-TFAHA) with improved selectivity and substrate efficiency to HDACs class IIa. The rationale for development of these radiotracers was based on previous reports that HDACs class IIa enzymes exhibit higher catalytic efficiency for Boc-L-Lys(ɛ-trifluoroacetyl)-MCA, as compared to Boc-L-Lys(ɛ-acetyl)-MCA. This is attributed to much higher electrophilicity of carbonyl carbon atom of the trifluoroacetyl moiety, as compared to acetyl moiety, despite the similarities in Van der Waals radii of acetyl and trifuloroacetyl moieties (28–30). However, Boc-L-Lys(ɛ-trifluoroacetyl)-MCA has demonstrated high substrate affinity also to HDAC8 (class I), which was discouraging in terms of its selectivity to HDACs class IIa. It is well established that the rim region in the active site of individual HDACs mediates the contact with capping groups of substrates (or inhibitors) and influences their affinity to individual HDACs [28]. Therefore, in this study we tested the hypothesis that a smaller sized capping group, such as an aniline moiety, may preserve high substrate affinity of 6-(trifluoroacetamido)-1-hexanoicanilide to HDACs class IIa, while reducing its substrate affinity to HDAC8 and other HDACs class I enzymes. Also, we assessed whether the number of fluorine atom substitutions in the acetyl moiety influences the substrate affinity and selectivity of mono-, di-, and tri- fluoroacetyl-hexanoicanilides to different HDACs.

Herein, we report the synthesis of DFAHA and TFAHA, as well as the radiosynthesis of [^18^F]DFAHA and [^18^F]TFAHA. We demonstrate that TFAHA exhibits significantly higher substrate affinity and selectivity to HDACs class IIa, especially to HDACs 4 and 5, as compared to FAHA and DFAHA. Although we have previously reported the results of ^18^F-FAHA PET/MRI imaging studies in rhesus macaques [16], the results of PET imaging studies in rats comparing [^18^F]FAHA, [^18^F]DFAHA, and [^18^F]TFAHA head-to-head are reported here for the first time.

## Results and Discussion

Several previously studied radiolabeled hydroxamite HDAC inhibitors, including [^125/131^I]-SAHA [29], [^11^C]MS-275 [30], [^18^F]SAHA [31], [^18^F]FESAHA [32], and [^64^Cu]CUDC-101 [33] demonstrated poor accumulation in the brain due to inability to efficiently cross the BBB. Other hydroxamite-based HDAC inhibitors containing more lipophilic capping groups, such as the adamantyl in [^11^C]martinostat, demonstrated efficient cellular membrane and BBB penetration of this radiotracer, as well as efficient visualization and quantification of HDACs class I expression levels in the brain and other organs in non-human primates [34]. Thus, PET imaging has been proven as an effective tool for image-guided optimization of potent BBB-permeable HDAC inhibitors (19, 35–37).

We focused on the development of class- and isoform- selective radiolabeled substrates instead of radiolabeled inhibitors, because of their ability to visualize not only the localization and magnitude of HDACs expression, but more importantly, their expression-activity product. In this paper, we report two novel radiolabeled substrate-based radiotracers [^18^F]DFAHA and [^18^F]TFAHA with enhanced enzyme selectivity for HDAC Class IIa, as compared to [^18^F]FAHA (14, 20). We demonstrate that increasing the number of fluorine atoms in the acetyl moiety from [^18^F]FAHA to [^18^F]DFAHA to [^18^F]TFAHA increases not only the selectivity and catalytic efficiency of these substrates for HDACs class IIa, but also improves the metabolic entrapment of radiolabeled leaving groups ([^18^F]difluoroacetate and [^18^F]trifluoroacetate) in the brain. In contrast, previously reported studies with [^11^C]6-acetamido-1-hexanoicanilide (^11^C-AHA) demonstrated significantly lower uptake and more uniform distribution in the brain, as compared to ^18^F-FAHA [19].

**Fig 1:**
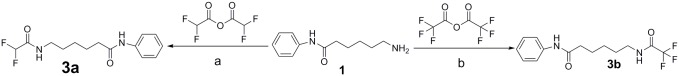
Synthesis of DFAHA and TFAHA. Reaction conditions are as follows: a) RT overnight; b) 2mL DCM, stirred overnight.

The syntheses of DFAHA (**3a**) and TFAHA (**3b**) are shown in Fig 1. Compound **1** was synthesized following a previously published method in 80% yield [35]. Compound **1** was the key intermediate for the synthesis of the precursor compounds for both DFAHA (**3a**) and TFAHA (**3b**). Reaction of **1** with bromofluoro acetyl chloride (freshly prepared by reacting bromofluoro acetic acid with thionyl chloride), and triethylamine in DCM at 0°C, produced compound **2a** in 15% yield. Due to the low boiling point of bromofluoro acetyl chloride, it was not possible to evaporate excess thionyl chloride prior to adding the compound **1**; for this reason, less than 1 equivalent of thionyl chloride was used in the reaction. Compound **2a** was obtained as a diastereoisomeric mixture and characterized by ^1^H, ^13^C and ^19^F NMR spectroscopy and high-resolution mass spectroscopy. The ^1^H NMR spectrum of compound **2a** showed 2 doublets corresponding to the fluorobromomethyl proton at 7.03 and 6.74 ppm with coupling constants of 49.45 Hz (typical for F-H coupling). Based on the relative integration of these two doublets the, diasteromeric mixture was obtained in an 80:20 ratio. The corresponding ^19^F-NMR spectrum showed 2 doublet peaks at -147.34 and -143.52 ppm with coupling constants 50.35 Hz and 48.83 Hz respectively, and the same diasteromeric ratio of 80:20. Resolving the diastereomeric mixture was unnecessary, because the subsequent reaction (radio-fluorination) led to the formation of difluroacetyl amide, which is optically inactive. The yield of this reaction is quite low, only 15%. As an alternative, we performed this reaction using the isobutyl chloroformate as intermediary step to aid the coupling. Also, we tested N,N'-dicyclohexylcarbodiimide (DCC) as an alternative coupling agent with 4-dimethylaminopyridine (DMAP) as a catalyst. Although not yet tested, other alternative coupling agents such as hydroxybenzotriazole (HOBT) or (1-[Bis(dimethylamino)methylene]-1H-1,2,3-triazolo[4,5-b]pyridinium 3-oxid hexafluorophosphate) (HATU) can be explored with N,N-diisopropylethylamine (DIPEA) as catalyst. One possible reason for the consistently low yield is the bromine atoms ability to couple with the amine of compound **1.** The competition for coupling site between the bromine and the chlorine will inherently lower the correct product formation. Reaction of compound **1** with difluorobromo acetyl chloride in DCM in the presence of triethylamine [36] produced the precursor, compound **2b**, in 50% yield. Consistent with the structure of **2b**, a singlet at -60.07 ppm was observed in the ^19^F NMR spectrum.

**Fig 2:**
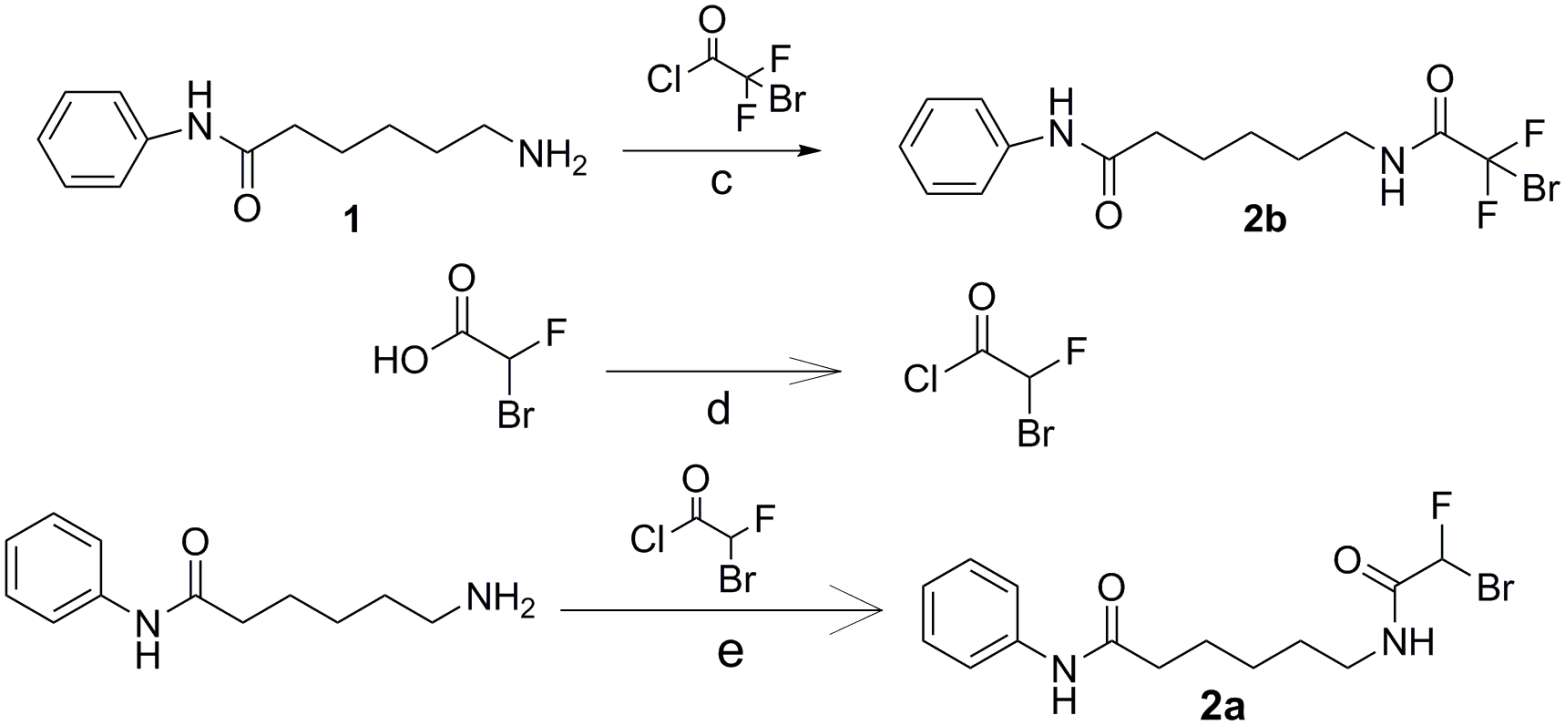
Synthesis of DFAHA and TFAHA precursors. Reaction conditions are as follows: c) pyridine, acetyl chloride added drop-wise at 0°C, stirred overnight at RT d) 0.9 eq. SOCl_2_, 12 hr. stirred under argon at 40°C, catalytic DMF; e) DCM, triethylamine added drop-wise at 0°C, stirred 24 hr. under argon at RT.

**Fig 3:**
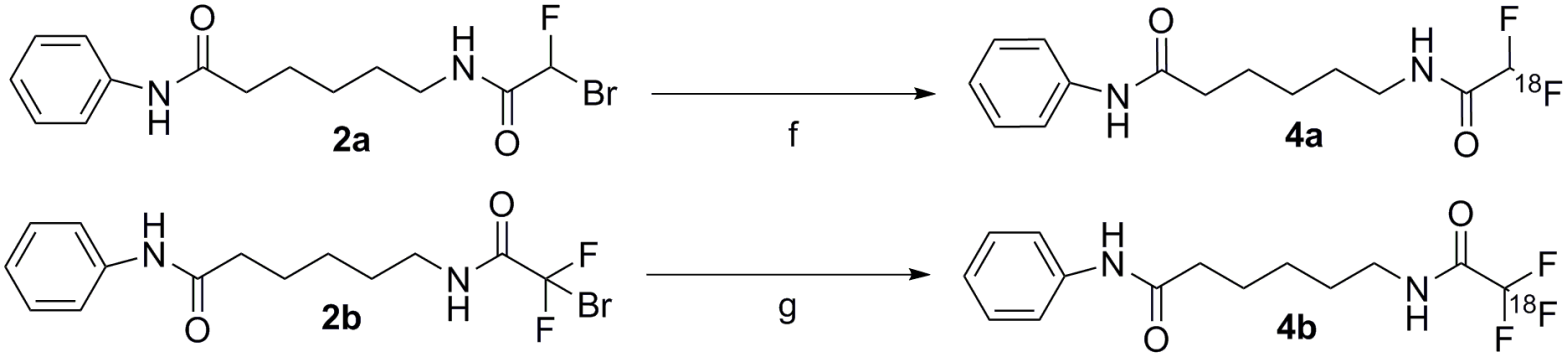
Synthesis of [^18^F]DFAHA and [^18^F]TFAHA. Reaction conditions are as follows: f) [^18^F]KF, K_2,2,2_; 0.4mL ACN, 105°C 25 min.; g)[^18^F]KF, K_2,2,2_; 0.4mL ACN, 105°C 25 min.

**Fig 4:**
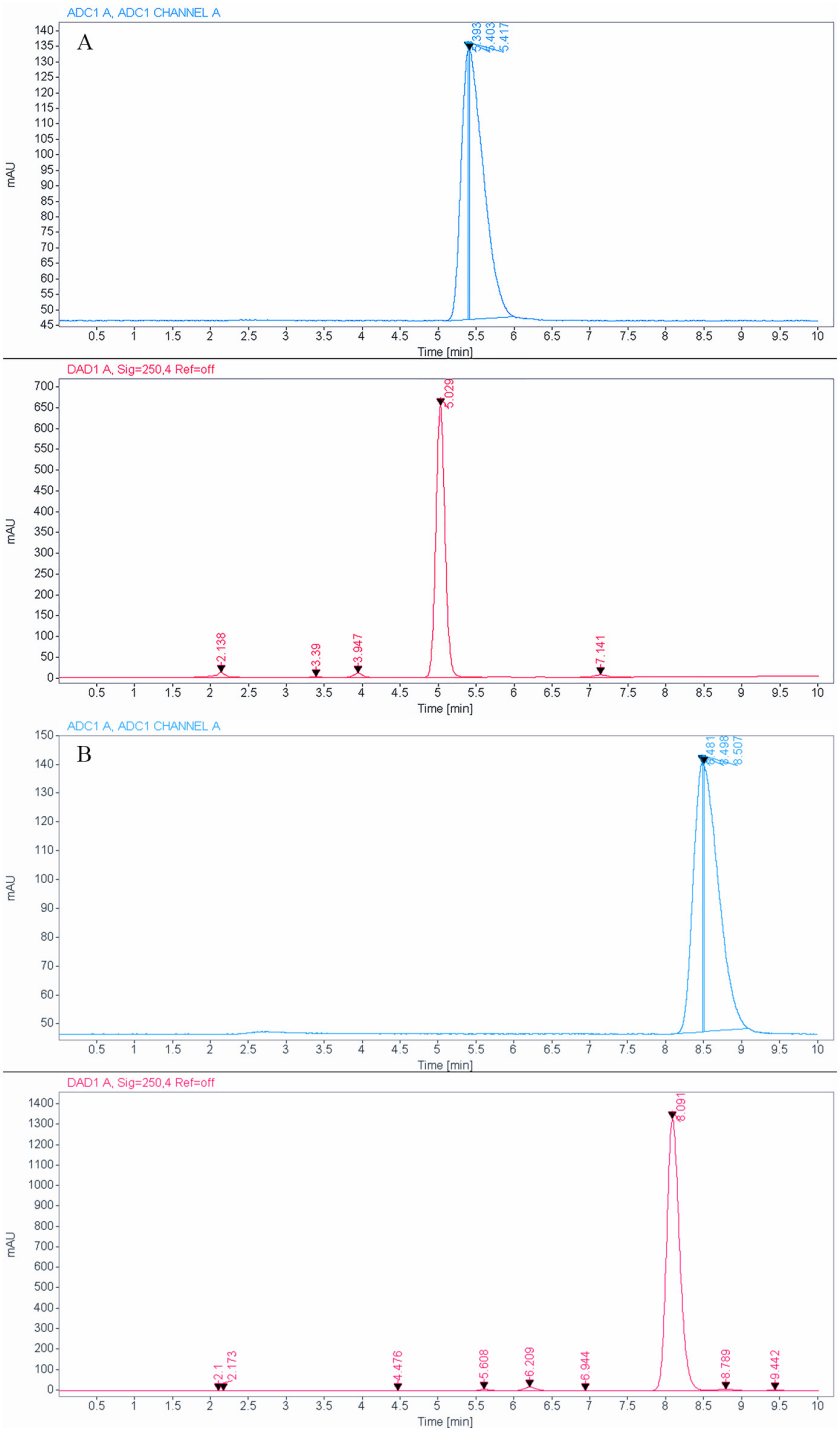
A: Shows Compound **4a** in blue co-injected with compound **3a**, the cold standard, in red. B: Compound **4b** shown in blue with compound **3b**, the cold standard, shown in red. The blue shows the clean radioactive spectrum, of the pure compound. UV detection was done at 254nm.

Compound **3a** was synthesized from **1** by reacting with difluoroacetic anhydride with no additional solvents in 42% yield. The ^1^H NMR spectrum of **3a** in DMSO-*d*_*6*_ was consistent with the structure and the geminal proton observed at 6.16 ppm as a doublet with a coupling constant of 53.8 Hz. The ^19^F NMR spectrum showed a doublet at -125.65 ppm with coupling constant 54.93 Hz, matching the coupling seen in the ^1^H NMR spectrum. Compound **3b** was obtained in 51% yield by reacting compound **1** with trifluoroacetic anhydride with only a small amount of DCM (~2 ml). A singlet fluorine peak was observed at -74.32 ppm in the ^19^F NMR spectrum of **3b**, which is consistent with the structure of three fluorines in the trifluoroacetyl moiety. Compounds **3a** and **3b** were used as non-radioactive standards for HPLC analysis and biochemical assays (Fig 2). Compounds **4a** and **4b** were synthesized in radiochemical yields of 25% and 22%, respectively (Fig 3). The identity of the radioactive product was confirmed by co-elution with nonradioactive standard as it is not possible to test the purity of radiochemical compounds by NMR. The purity of the compounds was greater than 95%, as assessed by analytical radio-HPLC, and specific activities ranging between 60 and 80 GBq/μmole. The quality control HPLC chromatograms taken for compounds **4a** and **4b** are shown in Fig 4a and 4b respectively.

**Fig 5:**
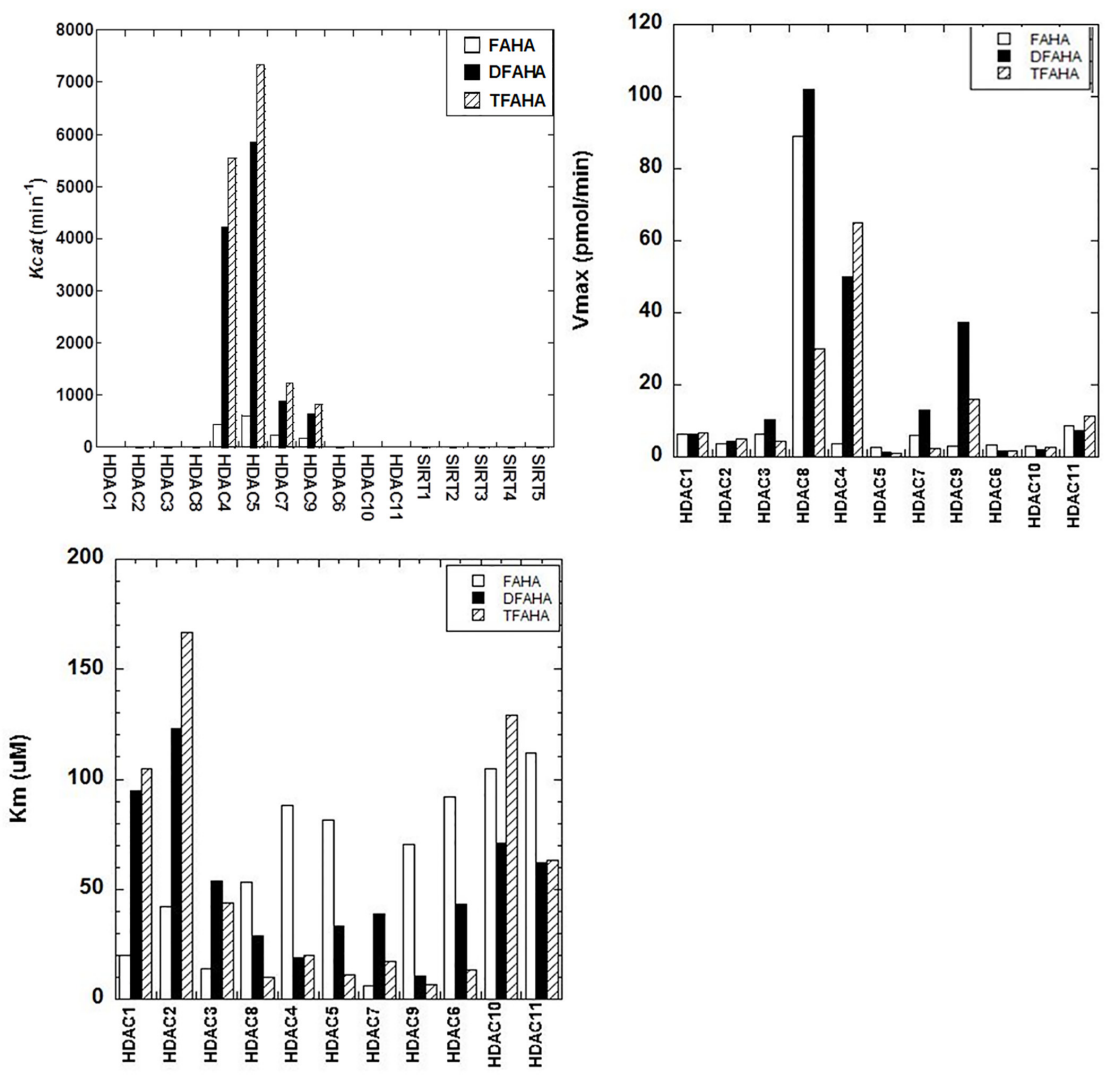
A) Substrate affinity of FAHA, DFAHA, and TFAHA to different recombinant HDACs *in vitro*. The substrate affinity is expressed as *Kcat*. B) Maximum catalytic rate of FAHA, DFAHA, and TFAHA for different recombinant HDACs *in vitro*, expressed as *v*_*max*_. C) The concentration required for half of the maximal catalytic activity for all recombinant HDACs expressed as *k*_*m*_.

Evaluation of substrate affinity of FAHA, DFAHA, and TFAHA in a panel of recombinant HDACs demonstrated the selectivity of these compounds for HDACs class IIa (HDACs 4, 5, 7, and 9), especially for HDACs 4 and 5. The k_cat_ values in HDACs class IIa were two orders of magnitude higher than those for other classes of HDACs (Fig 5a). The higher k_cat_ values of TFAHA, as compared to DFAHA and FAHA demonstrates the increasing ability of HDACs class IIa to cleave the trifluoro acetyl moiety, as compared to both difluoroacetyl and fluoroacetyl moieties. The HDAC class IIa enzymes also have lower *k*_*m*_ values and higher *v*_max_ values for TFAHA than the other HDACs enzymes (Fig 5b and 5c). There is a significant change in the *v*_*max*_ value of TFAHA from those of FAHA or DFAHA for HDAC8, demonstrating the decreased cleavage efficiency of HDAC class I with the addition of fluorine atoms (Fig 5b). Also, these results are consistent with previous reports demonstrating that HDACs class IIa enzymes exhibit higher catalytic efficiency for Boc-L-Lys(ɛ-trifluoroacetyl)-MCA, as compared to Boc-L-Lys(ɛ-acetyl)-MCA, which is not an efficient substrate to class IIa HDACs [37]. This explains, at least in part, why [^11^C]6-acetamido-1-hexanoicanilide (^11^C-AHA) demonstrated significantly lower uptake and more uniform distribution in the brain in the regions of high accumulation of ^18^F-FAHA [19].

**Fig 6:**
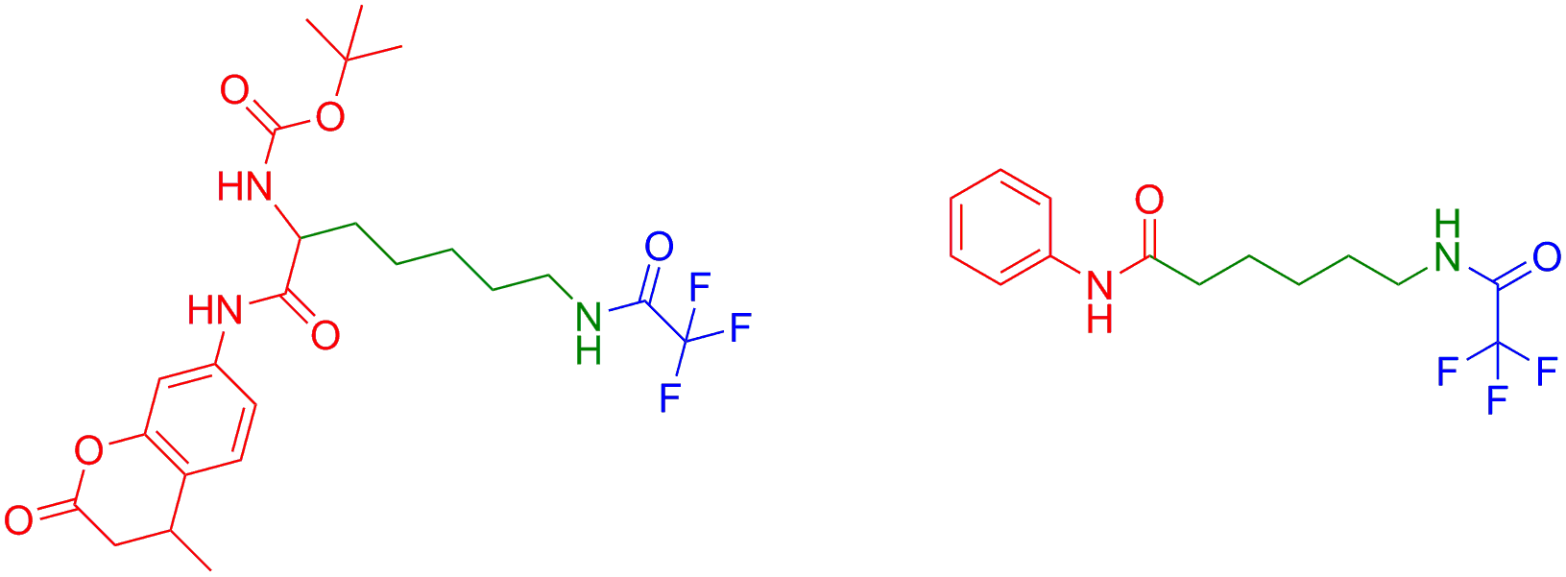
The three parts of a HDAC substrate, the cap (red), the linker (green), and the leaving group (blue).

It is important to note, that the entire structure of the substrate molecule plays a role in the selectivity for HDACs class IIa. The structure of HDAC substrates can be subdivided into three regions, which individually contribute to HDAC class and isoform selectivity: 1) the leaving group; 2) the linker; and 3) the capping moiety (Fig 6). Previous studies demonstrated that trifluoroacetyl group can be cleaved by HDAC 8 when it is attached to a lysine residue flanked by Boc and MCA (Boc-Lys(TFA)-MCA) [38]. In contrast, the phenyl capping group and lysine linker in DFAHA and TFAHA confer selectivity of these compounds only to HDAC Class IIa, with no significant affinity to HDAC8.

**Fig 7:**
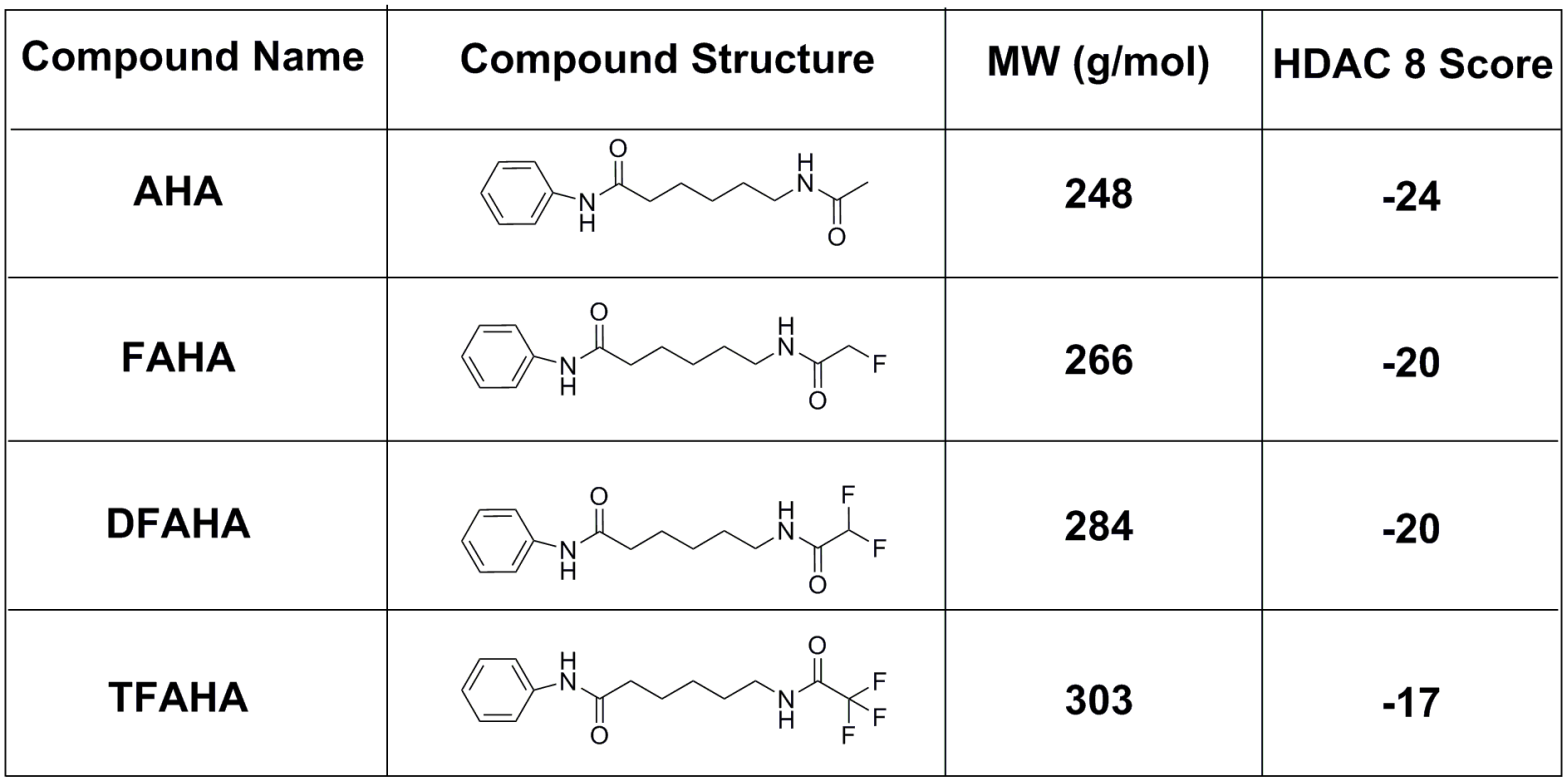
The structures of compounds and docking scores with HDAC8, a HDAC Class I enzyme. A lower docking score indicates higher affinity.

Structural comparison of active sites of HDACs class I and IIa enzymes may explain the mechanism of selectivity of FAHA, DFAHA and TFAHA for HDACs class IIa. The major differences between HDACs class I (1, 2, 3 and 8) and HDACs class IIa (4, 5, 7 and 9) is the substitution of His976 (class IIa) for Tyr306 (class I) in the catalytic site. This change in amino acid residues in the catalytic site results in negligible intrinsic deacetylase activity but has two important structural consequences: the change in size of the active site, and the removal of a hydrogen bond donor site. Class I HDACs have a smaller catalytic cavity than class IIa enzymes, which explains why larger leaving groups do not fit in the catalytic site and are not cleaved by these enzymes. The docking results for HDAC 8 (class I) shown in Fig 7, demonstrate that increasing the size of the leaving group (acetyl < trifluoroacetyl) increases the docking score, reflecting reduced ability of a molecule to fit inside the catalytic site of an enzyme. Indeed, previous studies have reported that larger leaving groups, such as propionyl, butyryl, (Z)-but-2-enyl, isobutyryl, 3-hydroxypropanyl, 3-methylbutanyl, 4-amino-4-oxobutanyl, and several other similar compounds are poor substrates for class I HDACs [39].

**Fig 8:**
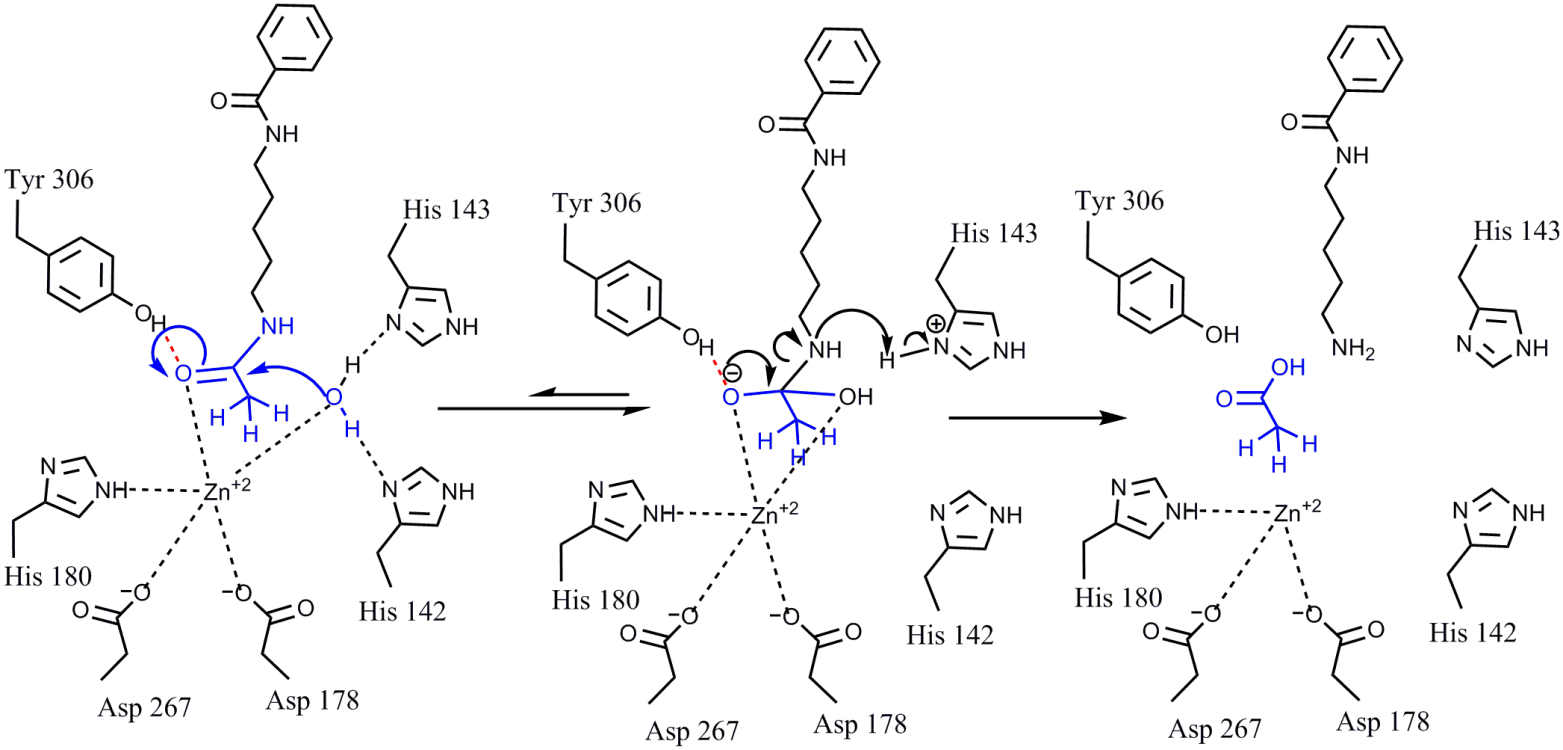
Mechanism of deactylation of AHA by HDAC8. In HDAC8 and other HDACs class I enzymes, the hydrogen bond forming between Tyr306 and the carbonyl oxygen of the acetyl moiety increases the electrophilicity of the carbonyl carbon, rendering it more susceptible to the nucleophilic attack by the activated water molecule, bound to His142 and His143. This leads to the formation of a tetrahedral oxyanion intermediate stabilized by Zn^2+^ ion and the hydroxyl group of Tyr306. Subsequently, the amide bond is cleaved and the acetyl moiety is released.

**Fig 9:**
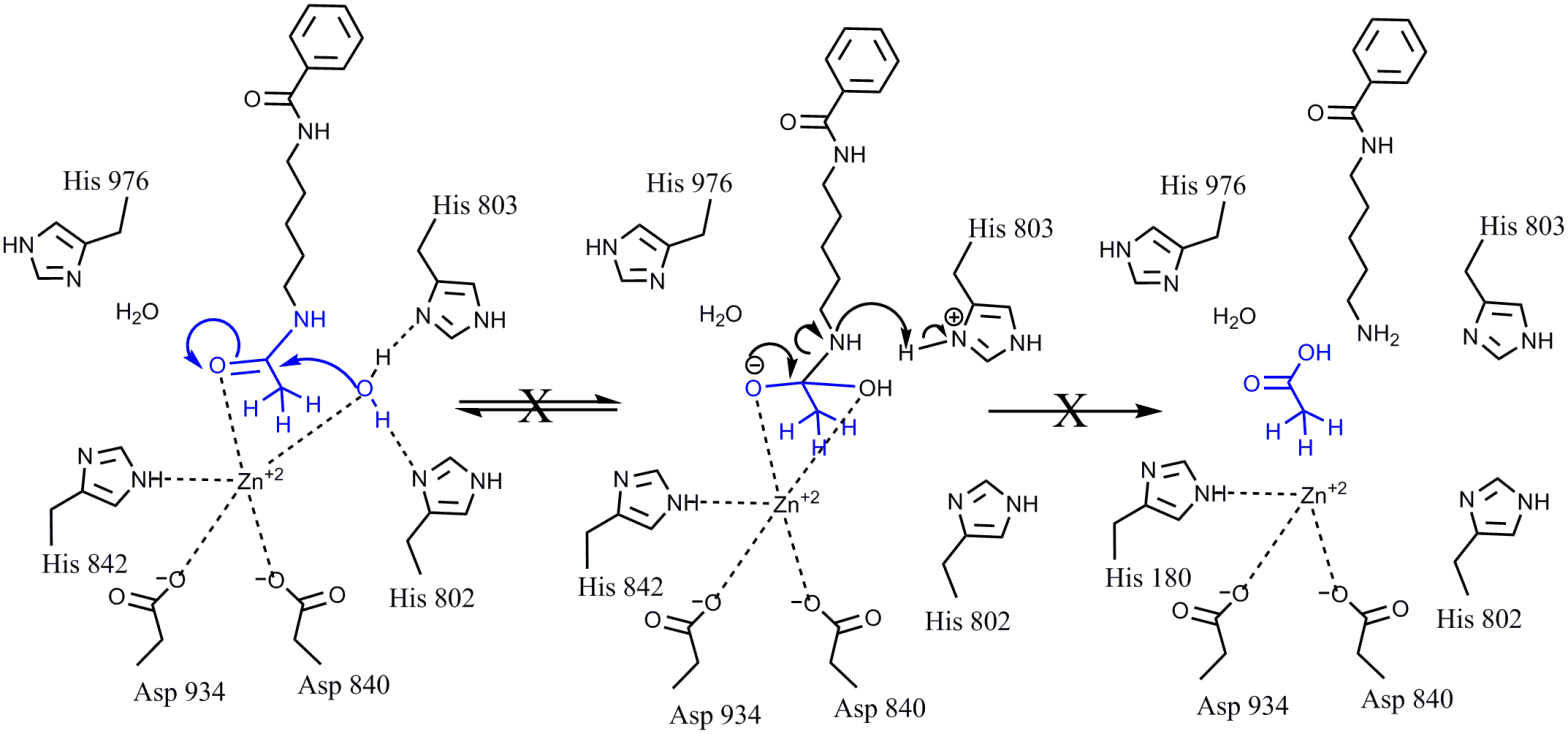
The mechanism of action of HDAC4. As compared to HDAC class I, HDAC class IIa enzymes exhibit significantly reduced ability to deacetylate. In class IIa enzymes the His976 located in the same position in the catalytic site as Tyr306 in class I HDACs does not serve as a hydrogen bond donor to bind to the carbonyl oxygen of the leaving acetyl group and thus reduces the susceptibility of carbonyl carbon to nucleophilic attack by the water, as in HDAC class I (see Fig 4).

**Fig 10:**
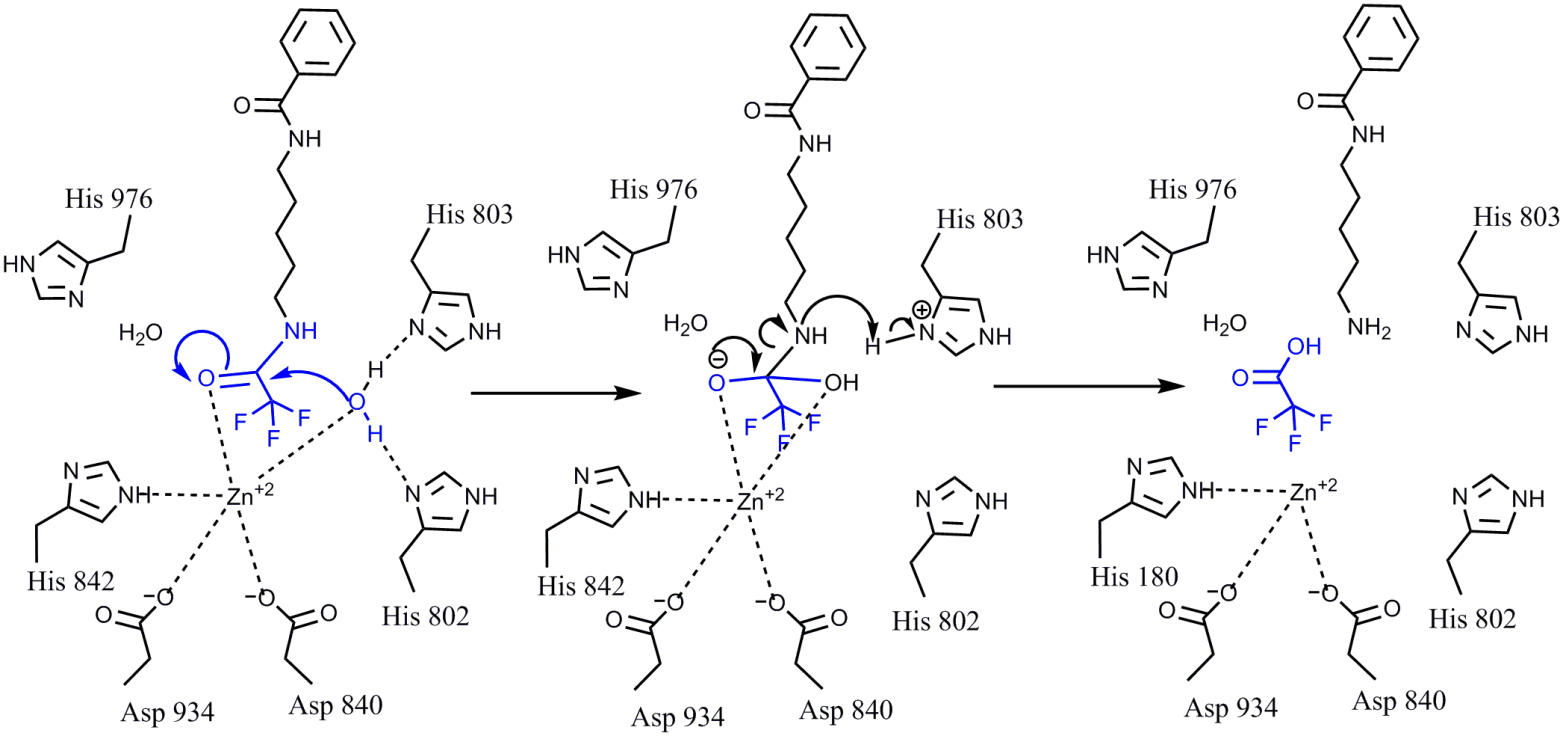
Proposed mechanism of de-trifluoroacetylation by HDAC4. Increased electronegativity of trifluoromethyl moiety of the trifluoroacetyl leaving group increases the susceptibility of the carbonyl carbon to the nucleophilic attack by the water molecule bound to His802 and His803 and enables the catalytic activity of HDACs class IIa. This results in formation of the tetrahedral oxyanion, which is irreversible, thereby releasing the trifluoroacetate.

HDAC class IIa enzymes lack a hydrogen bond donor that can bind to the carbonyl oxygen of the leaving acetyl group as a result of the substitution His976 (class IIa) for Tyr306 (class I), which renders the acetyl group inactive (Figs 8 and 9). In HDAC class I, this hydrogen bond increases the electrophilicity of the carbonyl carbon in the acetyl group, which renders it more susceptible to the nucleophilic attack by the activated water molecule, bound to His142 and His143. This leads to the formation of a tetrahedral oxyanion intermediate stabilized by the Zn^2+^ ion and by the hydroxyl group of Tyr306 of HDAC8. Consecutive replacement of the hydrogen atoms in the methyl moiety of the leaving acetyl group by fluorine atoms reduces the enzymatic activity in HDACs class I. This suggests that increasing the electronegativity of the leaving group is not sufficient to overcome the size increase, which may force the carbonyl carbon to rotate away from the activated water molecule, adding additional distortion to the hydrogen bonding with Tyr306. Increasing the electronegativity should increase the reactivity of carbonyl carbon, but since this is not the case, it is reasonable to assume that the relative increase in the effective size of the leaving group in TFAHA > DFAHA > FAHA may be a determining factor in the decreasing enzymatic activity. In contrast, increasing the number of fluorine atom substitutions in the methyl moiety of the acetyl leaving group increases the electrophilicity of the carbonyl carbon and restores the catalytic activity of HDACs class IIa by facilitating the nucleophilic attack on the carbonyl carbon of the acetyl moiety by activated water molecule bound to His802 and His803. As a result, the formation of the tetrahedral oxyanion is irreversible and releases trifluoroacetate as shown in Fig 10.

**Fig 11:**
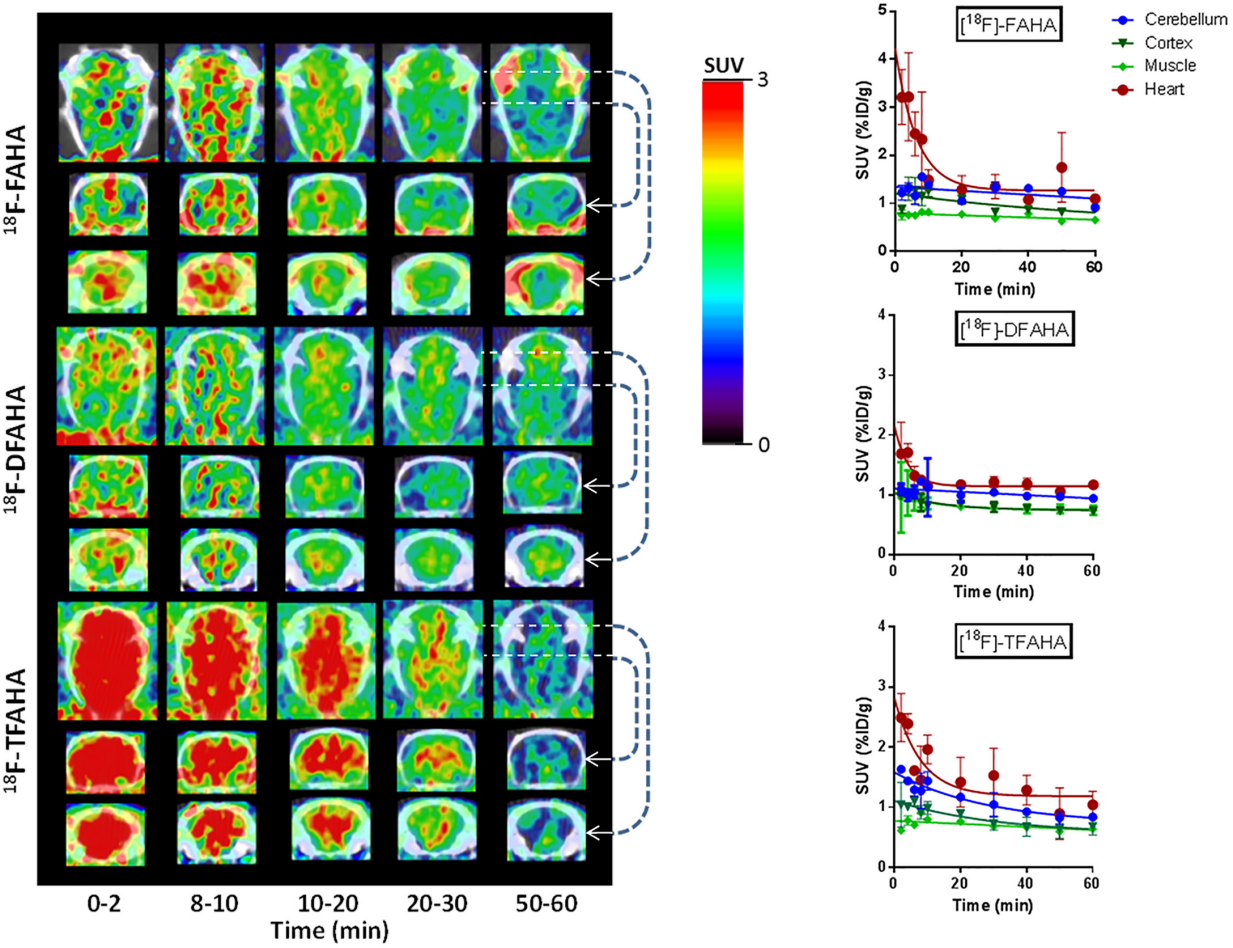
PET/CT images (A) of the spatial and temporal dynamics of influx, distribution, clearance and retention of each of the three radiotracers at different time intervals after intravenous administration: [^18^F]FAHA (top panel), [^18^F]DFAHA (middle panel), and [^18^F]TFAHA (bottom panel). In each panel, PET/CT images are provided in three different planes: top row—axial images through the middle of the brain; middle row–coronal images through the middle of the brain; bottom row—coronal images through the cerebellum area. Images are color-coded to range of standard uptake values (SUV) shown in the color bar and cross-normalized to facilitate direct comparison of radioactivity distribution. Corresponding time-activity plots (B) of each radiotracer are provided on the right hand side for: cerebellum (blue diamonds), muscle (green triangles), cortex (red squares), and heart (grey crosshairs).

The uptake, accumulation, and clearance of [^18^F]FAHA, [^18^F]DFAHA, and [^18^F]TFAHA in various regions of the rat brain were studied using dynamic PET/CT imaging. Higher levels of accumulation of all three radiotracers were observed primarily in *n*. *accumbens*, *hippocampus*, *amygdala*, *periaqueductal grey*, and *cerebellum* (Fig 11), where the HDACs 4 and 5 are abundantly expressed [16]. The alignment of the PET/CT images with the digital rat brain atlas [40] was used to identify regions of interest (Text A in S1 File; Fig A in S1 File). Highly selective accumulation of [^18^F]TFAHA-derived radioactivity was observed in the *cerebellum*, *n*. *accumbens*, *periaqueductal gray*, *and hippocampus* (dentate gyrus-CA1, CA3 region). The levels of [^18^F]TFAHA-derived radioactivity accumulation in these brain structures were higher than those from either [^18^F]DFAHA or [^18^F]FAHA. While [^18^F]FAHA exhibited moderate levels of radioactivity accumulation throughout the brain, including brain cortex, which is consistent with its higher substrate affinity to other HDAC classes, both [^18^F]DFAHA and, especially [^18^F]TFAHA, exhibited much lower accumulation in the cortex (Fig B in S1 File). Logan graphical analysis of [^18^F]FAHA, [^18^F]DFAHA and [^18^F]TFAHA accumulation in cerebellum using cortex as the reference tissue demonstrated that [^18^F]TFAHA was more actively accumulated (had a steeper slope of the Logan plot) in the cerebellar nuclei than either [^18^F]FAHA or [^18^F]DFAHA (Fig C in S1 File). Following PET/CT *in vivo* imaging with [^18^F]TFAHA, quantitative autoradiography (QAR) was performed in selected animals to verify the results at higher resolution of images. Fig D in S1 File demonstrates a high degree of correlation between the areas of ^18^F-TFAHA-derived accumulation displayed by autoradiography and the PET images shown in Fig A in S1 File. Higher levels of accumulation of [^18^F]TFAHA were observed using QAR in *n*. *accumbens*, *hippocampus*, *periaqueductal grey*, and in cerebellar nuclei.

Furthermore, our preliminary imaging studies demonstrated the feasibility of PET/CT with [^18^F]TFAHA for monitoring pharmacologic inhibition of HDACs class IIa with SAHA. A significant inhibition of [^18^F]TFAHA-derived radioactivity accumulation in the brain was observed when SAHA (100 mg/kg) was administered i.p. 30 min prior to i.v. administration of [^18^F]TFAHA (Fig E in S1 File). These results demonstrate that [^18^F]TFAHA is a much more selective and more efficient radiotracer for imaging expression-activity of HDAC class IIa enzymes in the brain, as compared to either [^18^F]DFAHA or [^18^F]FAHA.

In summary, current studies demonstrated that that [^18^F]TFAHA is a more selective and effective substrate-based radiotracer for non-invasive PET imaging of class IIa HDACs expression-activity in the brain. This novel PET imaging radiotracer can be readily translated into the clinic and may aid in the fundamental understanding of epigenetic regulation by HDACs class IIa involved in brain development and function, as well as in the mechanisms of different diseases, including traumatic brain injury and post-traumatic stress disorders, depression, drug addiction, Alzheimer’s, Huntington’s, brain tumors and other diseases of the brain. Furthermore, PET imaging with [^18^F]TFAHA should facilitate the development and clinical translation of novel HDACs class IIa selective inhibitors by enabling non-invasive quantification of their pharmacodynamics at the drug target level.

## Materials and Methods

### Reagents and Instrumentation

All reagents and solvents were purchased from Aldrich Chemical Co. (Milwaukee, WI), and used without further purification. Preparation of 6-amino-1-hexanoicanilide ***[41]*** was done following a previously published method [35]. Thin layer chromatography (TLC) was performed on pre-coated Dynamic Absorbance F-254 (Norcross, Georgia) silica gel, aluminum backed, plates. ^1^H, ^13^C and ^19^F NMR spectra were recorded on Mercury and Varian 400 MHz spectrometers at Wayne State University. High-resolution mass spectra (HRMS) were obtained from a Waters LCT Premier/XE mass spectrometer at the Lumigen Imaging Center in the Department of Chemistry at Wayne State University using electrospray ionization (ESI) technique. High performance liquid chromatography (HPLC) was performed with an 1100 series pump from Agilent Technologies (Stuttgart, Germany), with a built-in UV detector operated at 254 nm and a radioactivity detector with a single channel analyzer model FC3200 (NaI/PMT) detector from Eckert and Ziegler Radiopharma, Inc. (Germany) with an Ascentis RP-Amide column (Supelco, 4.4x150mm) for analytical and quality control chromatography. Semi-preparative HPLC was conducted on a Knauer P4.1S pump 10ml/Ti, with a Azura UVD 2.1S Detector using Clarity Software (Berlin, Germany) pump with an Alltima C18 (Fisher Scientific, 250x10mm) column with a UV detector at 254 nm and a radio detector model FC-3500 from Eckert and Ziegler AG (Berlin, Germany).

### Preparation of 6-(bromofluoroacetamido)-1-hexanoicanilide (2a)

Bromofluoro acetic acid (530mg, 3.3 mmol) was added to a reaction flask with 0.9 equivalents of thionyl chloride (210 μl, 2.9 mmol). The reaction was stirred for 4 hours at 60°C. Subsequently, the reaction mixture was cooled to room temperature and a solution of compound **1** (280 mg, 1.37 mmol) in 12 mL of anhydrous dichloromethane was added and followed by trimethylamine (2 ml, 15 mmol). The reaction was stirred under argon for 24 hours, after which, all of the solvent was evaporated and the compound was re-dissolved in dichloromethane and washed with aqueous saturated sodium bicarbonate solution. The organic layer was dried over sodium sulfate and evaporated. Flash column chromatography was used for purification of the compound with a gradient of solvent from 10% ethyl acetate in hexane to 50% ethyl acetate in hexane. Product **2a** (25 mg) was obtained in a 15% yield as an off-white solid.

Diastereomer 1: ^1^H NMR (DMSO-d_6_) δ: 9.83 (s, 1H, NH), 8.62 (s, 1H, NH), 7.56 (d, 2H, aromatic, *J* = 8.11 Hz), 7.26 (t, 2H, aromatic, *J* = 7.30 Hz), 6.99 (t, 1H, aromatic, *J* = 7.30 Hz), 7.03 (d, 1H, -CHFBr, J_gem_ = 49.45 Hz) 3.12 (q, 2H, CH_2_, *J* = 6.49 Hz), 2.27 (t, 2H, CH_2_, *J* = 7.34 Hz), 1.57 (m, 2H, CH_2_), 1.46 (m, 2H, CH_2_), 1.28 (m, 2H, CH_2_). ^13^C NMR (DMSO-d_6_) δ: 189.88, 171.63, 164.66 (d, -C = O-CF, J = 22.13 Hz), 139.70, 128.97, 123.34, 119.58, 85.93 (d, -CHFBr, J = 262.46 Hz), 36.67, 28.89, 26.21, 25.13 ppm. ^19^F NMR (DMSO-d_6_) δ: -147.34 (d, *J* = 50.35 Hz), -143.52 (d, J = 48.83). HRMS (m / z): [M + H]^+^ calculated for C_14_H_20_FBrN_2_O_2_, 367.0445; found 367.0433 (M+H).

Diastereomer 2: ^1^H NMR (DMSO-d_6_) δ: 9.83 (s, 1H, NH), 8.62 (s, 1H, NH), 7.56 (d, 2H, aromatic, *J* = 8.11 Hz), 7.26 (t, 2H, aromatic, *J* = 7.30 Hz), 6.99 (t, 1H, aromatic, *J* = 7.30 Hz), 7.03 6.74 (d, 1H, -CHFBr, J_gem_ = 49.45 Hz), 3.12 (q, 2H, CH_2_, *J* = 6.49 Hz), 2.27 (t, 2H, CH_2_, *J* = 7.34 Hz), 1.57 (m, 2H, CH_2_), 1.46 (m, 2H, CH_2_), 1.28 (m, 2H, CH_2_). ^13^C NMR (DMSO-d_6_) δ: 189.88, 171.63, 164.66 (d, -C = O-CF, J = 22.13 Hz), 139.70, 128.97, 123.34, 119.58, 85.93 (d, -CHFBr, J = 262.46 Hz), 36.67, 28.89, 26.21, 25.13 ppm. ^19^F NMR (DMSO-d_6_) δ: -147.34 (d, *J* = 50.35 Hz), -143.52 (d, *J* = 48.83). HRMS (m / z): [M + H]^+^ calculated for C_14_H_19_FBrN_2_O_2_, 367.0445; found 367.0433 (M+H).

### Preparation of 6-(bromodifluoroacetamido)-1-hexanoicanilide (2b)

Comound **1** (200 mg, 0.97 mmol) was dissolved in dimethylformamide (7.0 mL) with two equivalents of diisopropylethylamine and stirred at 0°C. 2-bromodifluoro acetyl chloride (0.2 mL, 2.2 mmol) was added drop-wise to the mixture. The mixture was subsequently stirred at 0°C for 1 hour then warmed to room temperature and stirred for an additional 3 hours under argon. After evaporation of solvent, the compound was purified using flash column chromatography with a gradient of 10–50% ethyl acetate/hexane as the eluent. Product **2b** (150 mg) was obtained after evaporation as a white solid with 50% yield. ^1^H NMR (DMSO-d_6_) δ: 9.83 (s, 1H, NH), 9.17 (s, 1H, NH), 7.54 (d, 2H, aromatic, *J* = 7.34 Hz), 7.34 (t, 2H, aromatic, *J* = 7.34 Hz), 6.99 (t, 1H, aromatic, *J* = 7.83 Hz), 3.15 (q, 2H, CH_2_, *J* = 6.36 Hz), 2.27 (t, 2H, CH_2_, *J* = 7.34 Hz), 1.58 (m, 2H, CH_2_), 1.49 (m, 2H, CH_2_), 1.28 (m, 2H, CH_2_). ^13^C NMR (DMSO-d_6_) δ: 171.42, 159.95 (t, -CO-CF_2_Br, *J* = 27.47 Hz), 139.71, 129.03, 123.21, 119.33, 112.21 (t, -CF_2_Br, J = 315.10 Hz), 36.70, 28.58, 26.40, 25.21 ppm. ^19^F NMR (DMSO-d_6_) δ: -60.07 (s). HRMS (m/z): [M +H]^+^ calculated for C_14_H_17_N_2_O_2_F_2_Br 363.0520, found 363.0515.

### Preparation of 6-(difluoroacetamido)-1-hexanoicanilide (DFAHA 3a)

Compound **1** (200 mg, 0.97mmol) was stirred in difluoroacetic anhydride at room temperature until fully dissolved. And then triethylamine (1 mL, 7.2 mmol) was added gradually until the pH of the solution reached a value close to 8. The reaction mixture stirred at room temperature overnight. The anhydride was removed under reduced pressure and the residue was purified by flash chromatography using 50% ethyl acetate in hexane. Product **3a** was obtained as a white powder in a 42% yield. ^1^H NMR (DMSO-d_6_) δ: 9.82 (s, 1H, NH), 8.75 (s, 1H, NH), 7.56 (d, 2H, aromatic, *J* = 7.83 Hz), 7.26 (t, 2H, aromatic, *J* = 7.34 Hz), 6.99 (t, 1H, aromatic, *J* = 7.34 Hz), 6.16 (t, 1H, -CHF_2_, *J*_*gem*_ = 53.8 Hz), 3.12 (q, 2H, CH_2_, *J* = 13.1, 6.7 Hz), 2.28 (t, 2H, CH_2_, *J* = 7.34 Hz), 1.57 (m, 2H, CH_2_), 1.46 (m, 2H, CH_2_), 1.27 (m, 2H, CH_2_). ^13^C NMR (DMSO-d_6_) δ: 171.42, 162.48 (t, -CO-CF2, J = 24.41 Hz), 139.71, 129.03, 123.21, 119.33, 108.98 (t, -CHF_2_, J = 246.44 Hz), 36.83, 28.74, 26.47, 25.18 ppm. ^19^F NMR (DMSO-d_6_) δ: -125.65 (d, *J* = 54.93 Hz). HRMS (m / z): [M + H]^+^ calculated for C_14_H_19_F_2_N_2_O_2_, 307.1234; found 307.1226 (M+H).

### Preparation of 6-(trifluoroacetamido)-1-hexanoicanilide (TFAHA 3b)

Compound **1** (100 mg, 0.48 mmol) was dissolved in trifluoacetic anhydride (1.0 g, 4.76 mmol) and 2mL of dichloromethane at 0°C. The reaction mixture was stirred under argon for 20 min and then 2 hours at room temperature overnight. The solvent was evaporated and the crude compound was purified by flash column chromatography using 30% ethyl acetate in hexane. After evaporation of the solvent, the product **3b** was obtained as white solid in 51% yield. ^1^H NMR (DMSO-d_6_) δ: 9.83 (s, 1H, NH), 9.4 (s, 1H, NH), 7.57 (d, 2H, aromatic, *J* = 8.8 Hz), 7.25 (t, 2H, aromatic, *J* = 7.83 Hz), 6.99 (t, 1H, aromatic, *J* = 6.85 Hz), 3.16 (q, 2H, CH_2_, *J* = 13.1 Hz, *J* = 6.7), 2.28 (t, 2H, CH_2_, *J* = 7.5 Hz), 1.57 (m, 2H, CH_2_), 1.49 (m, 2H, CH_2_), 1.27 (m, 2H, CH_2_). ^13^C NMR (DMSO-d_6_) δ: 171.61, 156.51(q, -CO-CF3, *J* = 71.6, 35.86 Hz), 139.83, 129.18, 123.52, 119.42, 116.45 (q, -CF_3_, *J* = 576.6, 288.40 Hz), 36.69, 28.47, 26.30, 25.09 ppm. ^19^F NMR (DMSO-d_6_) δ: -74.32 (s). HRMS (m / z): [M + H]^+^ calculated for C_14_H_18_F_3_N_2_O_2_, 303.1320; found 303.1312 (M+H).

### Radiosynthesis of 6-([^18^F]difluoroacetamido)-1-hexanoicanilide ([^18^F]DFAHA 4a)

A solution of K[^18^F]/kryptofix in acetonitrile (1 ml) was received and transferred into a crimped V-vial. The acetonitrile removed under a stream of argon at 105°C. A solution of **2a** (6–7 mg) in dry acetonitrile (0.4 mL) was added to previously dried K^18^F/kryptofix and the mixture was heated at 100°C for 20 minutes. The reaction was cooled and the mixture was passed through a silica gel cartridge (Alltech, 900 mg) and eluted with 30% methanol in dichloromethane (2.5 mL). After evaporation of the solvent under a stream of argon at 80°C, the mixture was re-dissolved in HPLC solvent and purified by semi-preparative HPLC using 40% ACN/buffer solution. The compound eluted at 9.7 minutes. The solvent was evaporated under reduced pressure and the final product was re-dissolved in saline for animal injection. The product purity and identity was confirmed by co-injection with an authentic non-radiolabelled (**4a)** using an analytical HPLC. The compound was obtained in 25% decay corrected yield, with > 95% purity, and specific activity of 60–70 GBq/μmole.

### Radiosynthesis of 6-([^18^F]trifluoroacetamido)-1-hexanoicanilide ([^18^F]TFAHA 4b)

A solution of kryptofix/ K[^18^F] in acetonitrile (1 ml) was received and transferred into a crimped V-vial. The acetonitrile removed under a stream of argon at 105°C. A solution of **2b** (6–7 mg) in dry acetonitrile (0.4 mL) was added to previously dried K^18^F/kryptofix and the mixture was heated at 100°C for 20 minutes. The mixture was heated and stirred at 110°C for 25 minutes. The reaction was cooled and the mixture was passed through a silica gel cartridge (Alltech, 900 mg) and eluted with 30% methanol in dichloromethane (2.5 mL). After evaporation of the solvent under a stream of argon at 80°C, the mixture was re-dissolved in HPLC solvent and purified by semi-preparative HPLC using 40% ACN/buffer solution. The compound was eluted at 20 minutes. The solvent was evaporated on a high vacuum pump and the desired product was re-dissolved in saline for animal injection. The solvent was evaporated under reduced pressure and the final product was re-dissolved in saline for animal injection. The product purity and identity was confirmed by co-injection with an authentic non-radiolabelled (**4b)** using an analytical HPLC. The product was obtained in a 22% decay corrected yield, with > 95% purity, and specific activity of 70–80 GBq/μmole.

### In vitro HDAC enzyme affinity assay

*In vitro* enzyme assays were performed for a panel of Class I, II, III, and IV HDACs. 10 nmol solution of each test compound or 2 nmol of control substrate 2A (BPS Bioscience, San Diego, CA) was incubated in 100 μl of the HDAC assay buffer (25 mM Tris-HCl, pH 8.0, 137 mM NaCl, 2.7 mM KCl, 1 mM MgCl_2_) in the presence or absence of 1μg/ 100 μl of different HDAC enzymes (BPS Bioscience, San Diego, CA) at 37°C for 60 min. The reaction was quenched by heating the assay at 95°C for 3 min. Subsequently, 25 ul of the reaction mixture was analyzed by analytical HPLC (Agilent, Santa Clara, CA) equipped with Econosil C18 column (Altech Associates, Deefield, IL) using a mobile phase: 50% acetonitrile / 20 mM ammonium acetate, pH = 8.5. Detection of reaction products was detected via UV detection at 240 nm. The area under the peak corresponding to hexanoic anilide reaction product were integrated and expressed as percent of the area under the peak of the parent compound and converted into mass (moles). The K_cat_ parameters were determined as described elsewhere [42] using software GraphPad Prism 4 (GraphPad Software, La Jolla, CA).

### In silico modeling

The crystal structures 1T69 and 2VQJ were used for the docking studies of HDAC8 and HDAC4 respectively. For HDAC8 the missing residues 1–13 and 85–90 were added using MODELLER (UCSF, San Francisco, CA) [43]. The residues 85–90 are involved in the formation of a loop that is in close proximity to the inhibitor binding region of HDAC8. Due to this potential importance, the loop formed by these MODELLER-added residues was further refined using Chimera (UCSF, San Francisco, CA) [44]. The two crystal structures were hydrogenated using MolProbity software (Duke University, Durham, NC) [45]. Docking calculations were performed using the FlexX algorithm [46] as implemented in the LeadIT software package (BioSolveIT GmbH, Germany) [47]. For both HDAC8 and HDAC4, native ligands in the crystal structure (namely SHH for HDAC8 and TFG for HDAC4) were used as reference ligands for the docking calculations. A grid volume that covered amino acids within 20Å from each reference ligand was used. All the waters within the grid volume were taken as freely rotatable and displaceable. Pharmacophore rules based on the knowledge of ligand-receptor binding were used to guide the ligands towards the binding site. For both HDAC8 and HDAC4 docking was performed with the ligands AHA, FAHA, DFAHA, and TFAHA. Top 20 poses based on the docking score of each docking run were saved and the top 10 poses were used for binding affinity calculations using Hyde software (BioSolveIT GmbH, Germany) [48,49].

### PET Imaging Procedures in Animals

All studies involving animals were performed under a protocol (A 6-13-13) approved by the Institutional Animal Care and Use Committee of Wayne State University. Sprague-Dawley rats (200–250 g, N = 3) were anesthetized with 3% isoflurane in oxygen and maintained at 2% isoflurane in oxygen throughout the imaging studies. The body temperature was maintained using electronically-controlled heating pad (M2M Imaging, Cleveland, OH) set at 37°C. Anesthetized rats were placed in the microPET R4 scanner (Siemens, Knoxville, TN) in the supine position with the long axis of the animal parallel to the long axis of the scanner with the brain positioned in the center of the field of view. Each radiotracer (300–500 μCi/animal) was administered in saline via the tail-vein injection in a total volume ≤1.25 ml. Dynamic PET images were obtained over 60 minutes, followed by 2 overlapping frames (5 min each) acquired to obtain a whole body images of radiotracer biodistribution in other organs and tissues. After PET imaging, the positioning bed with the affixed anesthetized animal was transferred to the Inveon SPECT/CT scanner (Siemens, Knoxville, TN) and CT images and 4 overlapping frames (2 min each) were acquired covering the whole body using X-ray tube settings of 80 kV and 500 uA.

### Quantitative Autoradiography

After PET imaging (or at certain time point after 300–500 μCi radiotracer injection i.v.), the animals were sacrificed, the brain was rapidly extracted, frozen, and embedded in the mounting medium M1 (Shandon-Lipshaw, Pittsburg, PA). Serial 20 μm thick coronal sections of frozen brain tissue were obtained at -13°C using a cryomicrotome CM3050S (Leica, Germany). Tissue sections were thaw-mounted on poly-A lysine coated glass slides and heat-fixed for 5 min at 65°C on a slide warmer (Fischer Scientific, PA). For QAR, tissue sections were exposed to the phosphor plate (Fujifilm Life Science, Woodbridge, CT) along with set of 20 μm autoradiographic standards of known 18F radioactivity concentration, freshly prepared using calf liver homogenate. Knowing the radioactivity concentration in standards, the injected dose, and optical densities of each reference standard, a standard curve was developed, based on which the autoradiographic images were converted to color-coded parametric images of percent injected dose/g tissue (%ID/g) and/or standard uptake values (SUV) using software MCID 7.0 (Interfocus Imaging Ltd., Cambridge, UK).

### Image Analysis and Quantification

PET images were reconstructed using ordered subset expectation–maximization [50] method. PET image analysis was accomplished using the AMIDE software. Digital Rat Brain Atlas was used for alignment and identification of specific anatomical markers in the brain [51]. GraphPad Prism 6 (Graph Pad Software La Jolla, CA) and Excel 2010 (Microsoft, Redmond, WA) were used for image data analysis. Levels of accumulation of individual radiotracers in tissues were expressed as standard uptake values (SUV) that were calculated for the regions of interest [52] using the AMIDE software. The SUV is defined as the ratio of the tissue radioactivity concentration C (e.g. expressed as Bq/g tissue) at given time point post injection T, and the injected dose (e.g. in Bq, decay-corrected to the same time T), and normalized by the body weight in grams. Formulas for calculations for Logan plot analysis are provided in the supplemental materials [53,54].

## Supporting Information

S1 File Text A in S1 file. Additional Methods and Results. Fig A in S1 file. PET/CT images obtained with [^18^F]TFAHA at 30 min post radiotracer administration and The corresponding stereotactic maps for localization of signals from [^18^F]TFAHA-derived radioactivity. Fig B in S1 file. The average calculated SUV for each of the ROI’s and reference tissues for all three compounds. The error bars represent the standard deviation of the data. The stars indicate statistical significance obtained by two-way ANOVA with P < 0.01. Fig C in S1 file. The Logan plot for cerebellum with cortex used as a reference tissue. The stars represent statistical significance as determined by comparison of linear regression fit with a P < 0.01. Fig D in S1 file. Quantitative autoradiagraphy for ^18^F-TFAHA in the rat brain following *in vivo* i.v. injection of the radiotracer and PET imaging. The rat brain atlas maps are shown with the images for co-registration. Fig E in S1 file. PET/CT images of the same rat brain obtained at 30 min post [^18^F]TFAHA administration at (A) baseline and (B) after pretreatment with SAHA (100 mg/kg i.p. 30 min prior to injection of [^18^F]TFAHA).
(DOCX)
