# Supplementary material for: Novel Histone Deacetylase Class IIa Selective Substrate Radiotracers for PET Imaging of Epigenetic Regulation in the Brain
Source: PLoS One. 2015 Aug 5;10(8):e0133512. doi: 10.1371/journal.pone.0133512 (PMC4526562; doi:10.1371/journal.pone.0133512)
Supplement: S1 File — Additional Methods and Results. Fig A in S1 file. PET/CT images obtained with [18F]TFAHA at 30 min post radiotracer administration and The corresponding stereotactic maps for localization of signals from [18F]TFAHA-derived radioactivity. Fig B in S1 file. The average calculated SUV for each of the ROI’s and reference tissues for all three compounds. The error bars represent the standard deviation of the data. The stars indicate statistical significance obtained by two-way ANOVA with P < 0.01. Fig C in S1 file. The Logan plot for cerebellum with cortex used as a reference tissue. The stars represent statistical significance as determined by comparison of linear regression fit with a P < 0.01. Fig D in S1 file. Quantitative autoradiagraphy for 18F-TFAHA in the rat brain following in vivo i.v. injection of the radiotracer and PET imaging. The rat brain atlas maps are shown with the images for co-registration. Fig E in S1 file. PET/CT images of the same rat brain obtained at 30 min post [18F]TFAHA administration at (A) baseline and (B) after pretreatment with SAHA (100 mg/kg i.p. 30 min prior to injection of [18F]TFAHA). (DOCX) [file pone.0133512.s001.docx]

**Novel Histone Deacetylase Class IIa-Selective Substrate Radiotracers for PET Imaging of Epigenetic Regulation in the Brain**

Robin Bonomi^1^, Uday Mukhopadhyay^5^, Aleksander Shavrin^1^, Hsien-Hsien Yeh^6,7^, Anjoy Majhi^1^, Sajeewa Dewage^3^, Amer Najjar^4^, Xin Lu^1^, G. Andrés Cisneros^3^, William P. Tong^4^, Mian M. Alauddin^4^, Ren-Shuan Liu^6,7^, Thomas J. Mangner^2^, Nashaat Turkman^1^, Juri G. Gelovani^1^*,

**Quantification of dynamic PET imaging by multi-graphical analysis**

To locate the exact ROI’s the Rat Brain Atlas was referenced and the corresponding maps to PET images are shown in **Fig. A in S1 file**. While exact co-localization was difficult because the animal was not perfectly planar, these images show a very close representation of the sites for metabolite accumulation of the radiotracer. While the primary ROI of this paper is the cerebellum, the other regions of specific uptake are quantified and shown in **Fig. B in S1 file.**

We used the heart-derived blood time-activity curve (TAC) as an input function between 0 and 30 min, because a previous study showed that blood radioactivity during the first 30 min post-i.v. injection of [^18^F]FAHA is predominantly caused by the parent un-metabolized compound.


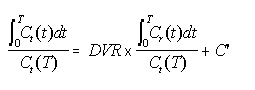
The dynamic PET imaging data were analyzed using Logan graphical analysis (25) to determine whether [^18^F]TFAHA PET/CT imaging could have highest substrate affinity for HDAC 4 and 5. The ratio of integrated radioactivity concentration in ROIs over time normalized by the radioactivity concentration at a given time point in tumor radioactivity concentration was set as the y axis. The ratio of integrated reference tissue radioactivity concentration over time normalized by tumor radioactivity concentration was set as the x axis of a Logan plot. The cortex was used as reference tissue, because relatively it has low HDAC4 and 5 activity or expression in the brain. The slope of the linear portion of the Logan plot is the distribution volume ratio (DVR). The slope of the linear portion of the lot is calculated as (**Eq. 2**). The binging potential (BP) can be calculated as **Eq. 3**. For these calculations the target ROI used is the cerebellum. The y-intercept is denoted as C’ and used to accommodate for differences in the immediate tracer uptake by tissue.

^18^F-FAHA has non-specific interactions to many other HDAC enzymes in the brain. Therefore, it shows a higher level of accumulation. The specific interaction, however, is actually much higher for TFAHA and DFAHA because these have a very similar uptake but act on far fewer enzymes. These conclusions are supported by the biochemical results showing that FAHA is a substrate for many of the HDAC enzymes in all four classes. The plot in **Fig. C in S1 file** is the Logan plot given by **Eq. 2** show the uptake for [^18^F]FAHA is very similar to that of [^18^F]DFAHA, while [^18^F]TFAHA uptake has a steeper slope and therefore higher levels of metabolite accumulation in the cerebellum.

Following PET/CT *in vivo* imaging with [^18^F]TFAHA, quantitative autoradiography (QAR) was performed to verify the results at higher resolution. **Fig. D in S1 file** demonstrates a high degree of correlation between the areas of ^18^F-TFAHA-derived accumulation displayed by autoradiography and the PET images in **Fig**. **A in S1 file.** Higher levels of accumulation of [^18^F]TFAHA were observed in n. accumbens, hippocampus, periaqueductal grey, and cerebellar nuclei.

Furthermore, our preliminary imaging studies in a rat demonstrated the feasibility of PET/CT with [^18^F]TFAHA for monitoring pharmacologic inhibition of HDACs class IIa with SAHA. Significant inhibition of [^18^F]TFAHA-derived radioactivity accumulation in the brain was observed when SAHA (100 mg/kg) was administered i.p. 30 min prior to i.v. administration of [^18^F]TFAHA (**Fig. E in S1 file**). Additionally, we are currently conducting expanded studies in rats to determine the IC50 values for SAHA for different regions of the brain.

**Fig. A** The corresponding map and brain image for co-localization and ROI identification.


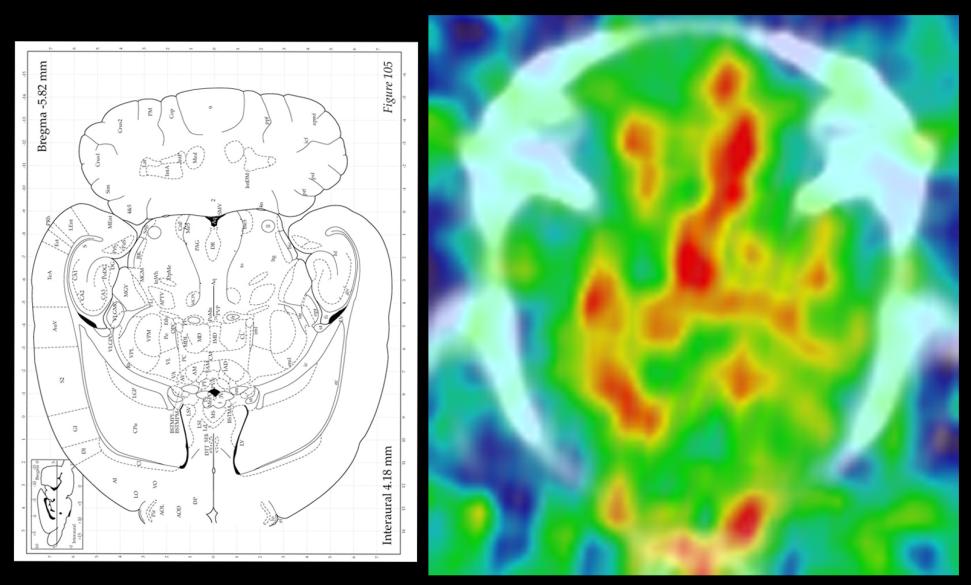


**SUV**

**0 -**


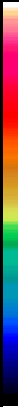


**2 -**


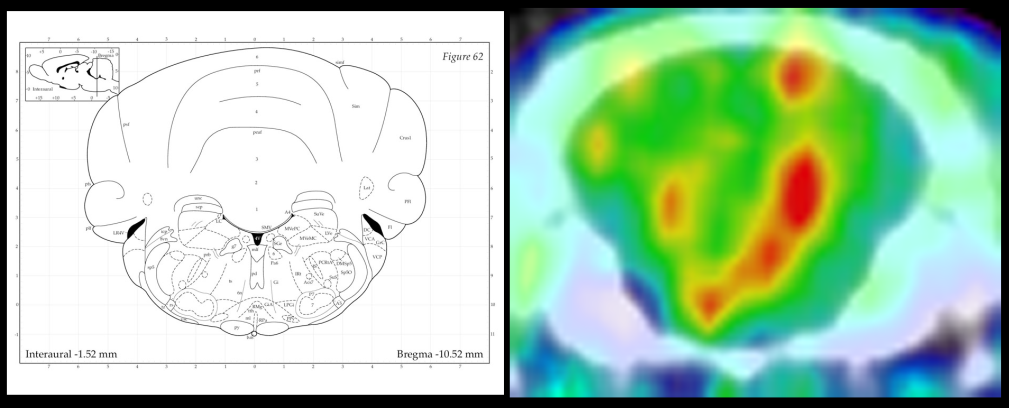


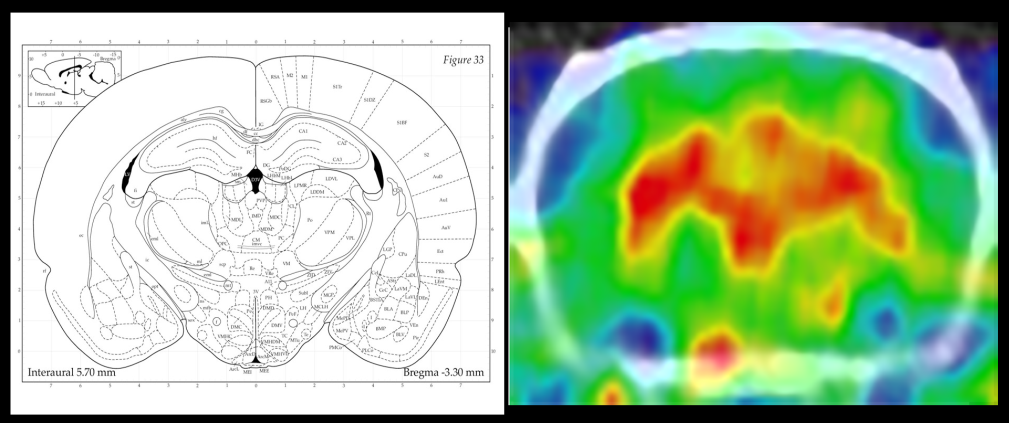

**Fig**. **B** The average calculated SUV quantification for each of the ROI’s and reference tissues for all three compounds. The error bars represent the standard deviation of the data. The stars indicate statistical significance obtained by two-way ANOVA with P < 0.01.

ʃ

ʃ

**Fig. C** The Logan plot for the primary ROI, cerebellum, with the cortex used as a reference tissue. The stars represent statistical significance as determined by comparison of linear regression fit with a P < 0.01


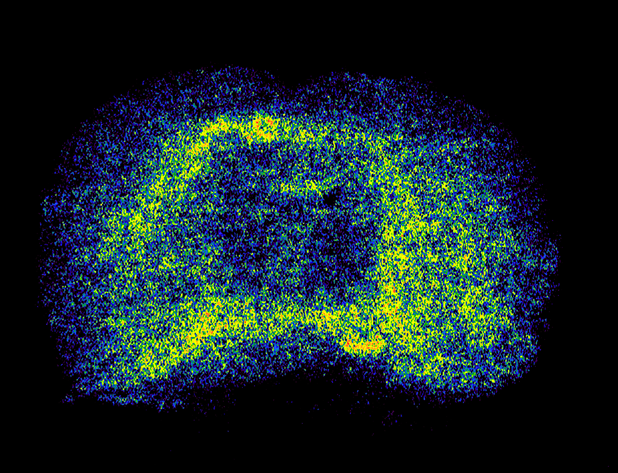

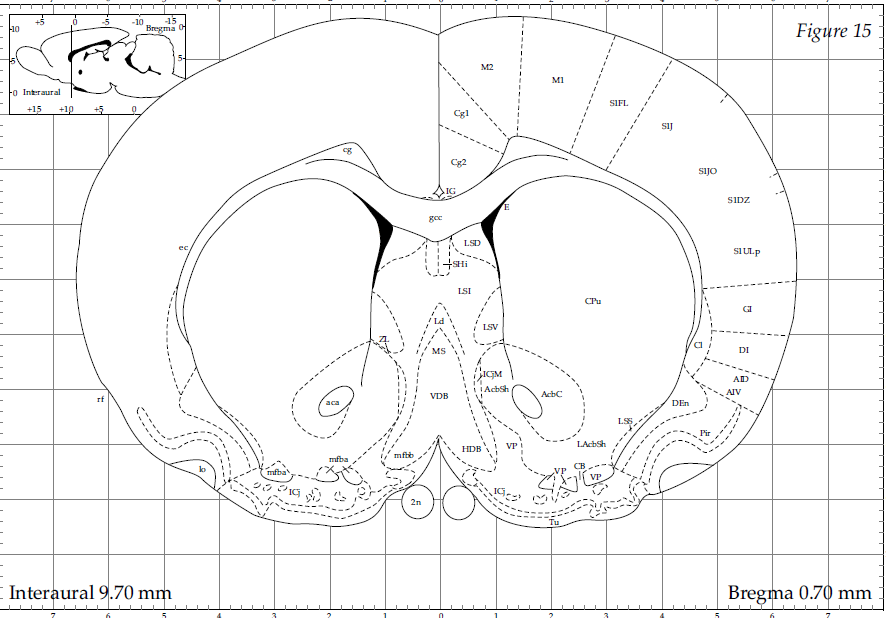

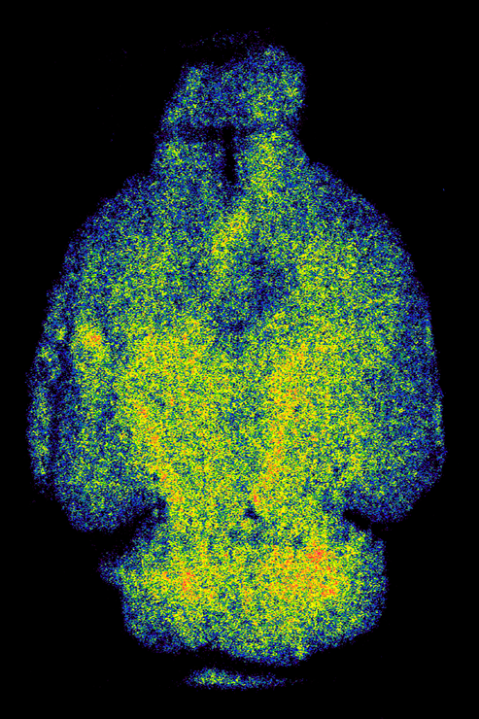

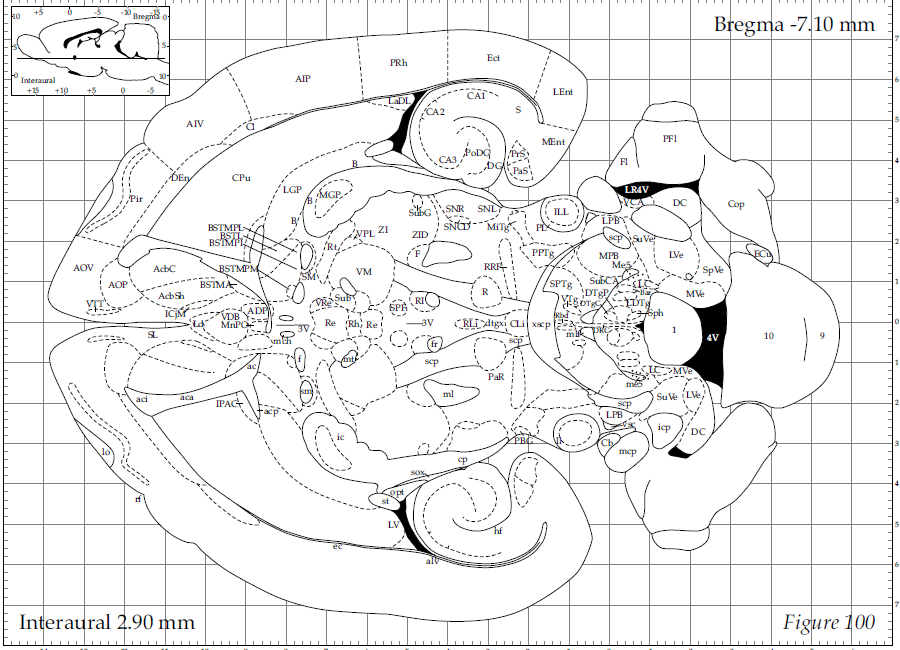

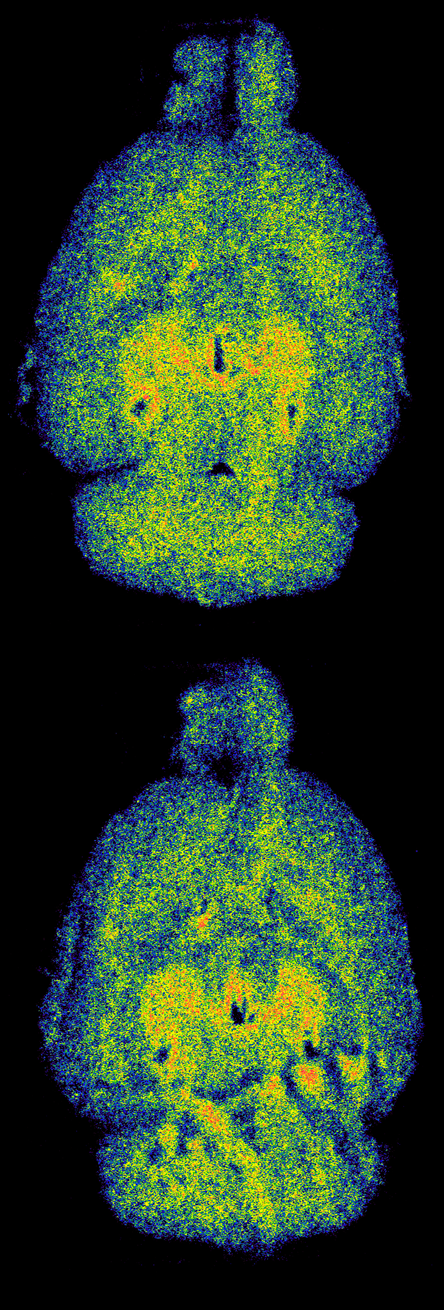

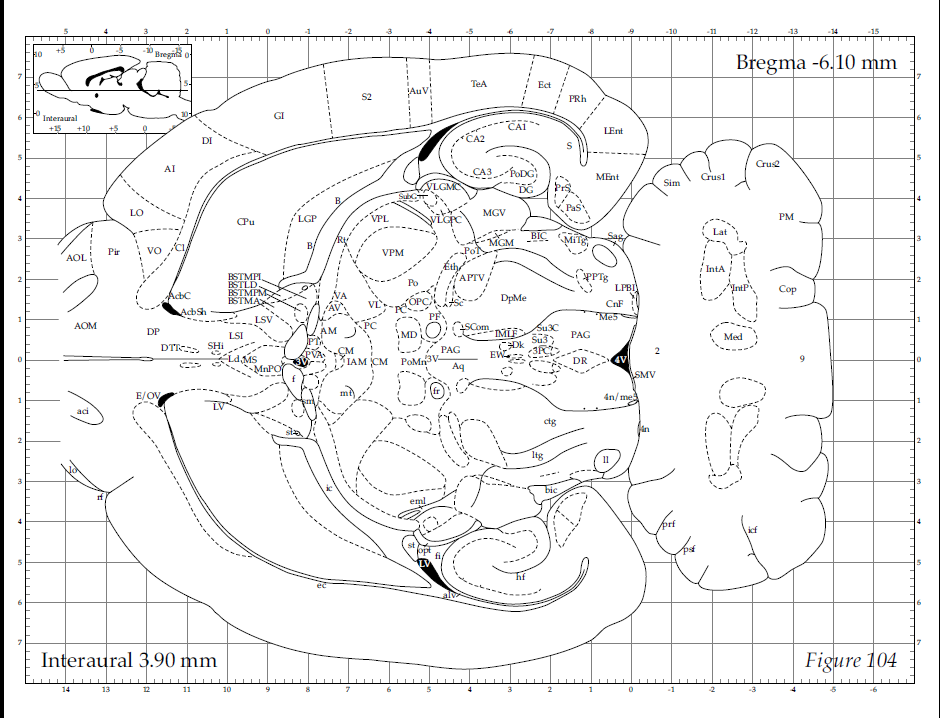

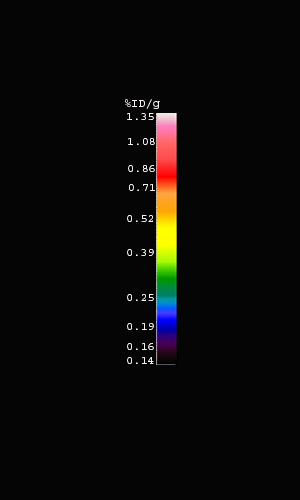


**2-**

**0-**

**SUV**

**Fig. D** Quantitative autoradiagraphy for ^18^F-TFAHA in the rat brain following *in vivo* i.v. injection of the radiotracer and PET imaging. The rat brain atlas maps are shown with the images for co-registration.


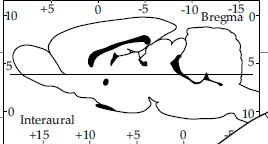


**A**


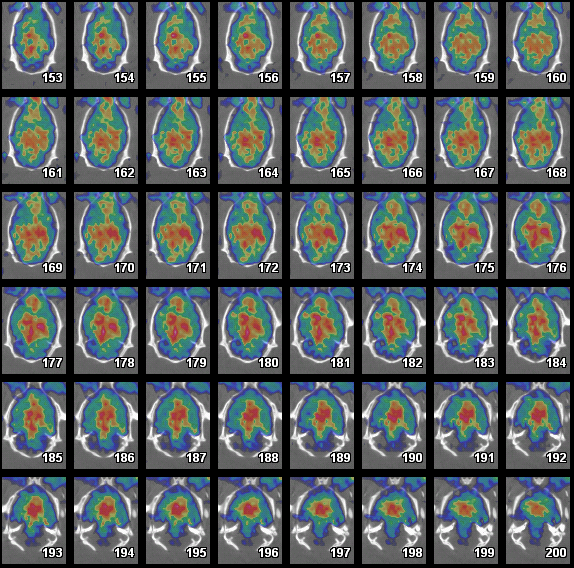


**SUV**

**0 -**


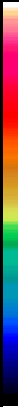


**2 -**

**B**


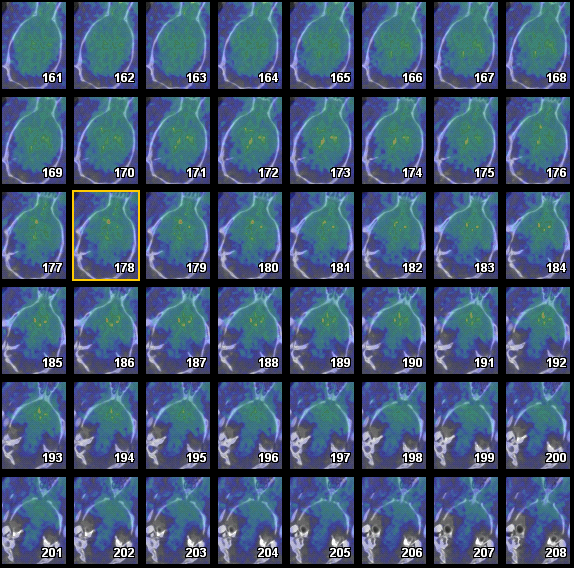


**Fig. E** PET/CT images of the same rat brain obtained at 30 min post [^18^F]TFAHA administration at (A) baseline and (B) after pretreatment with SAHA (100 mg/kg i.p. 30 min prior to injection of [^18^F]TFAHA).
